# Editorial: The effect of mycobiomes on health of forest trees

**DOI:** 10.3389/fmicb.2023.1162198

**Published:** 2023-02-27

**Authors:** Eeva Terhonen, Funda Oskay, Risto Kasanen

**Affiliations:** ^1^Forest Health and Biodiversity, Natural Resources Institute Finland (Luke), Helsinki, Finland; ^2^Department of Forest Entomology and Protection, Faculty of Forestry, Çankiri Karatekin University, Çankiri, Türkiye; ^3^Department of Forest Sciences, University of Helsinki, Helsinki, Finland

**Keywords:** mycobiome, forest trees, resilience, climate change, invasive pathogens

The unseen partners of forest trees such as filamentous fungi, bacteria and viruses contribute to the hidden biodiversity of forests. Shifts in environmental conditions driven by climate change together with biotic disturbance agents challenge the resilience of forests. This is frequently attributed to the unpredictable behavior of invasive fungal pathogens under persistent abiotic stress on the host. In this scenario, the importance of tree microbiome associations to either environmental adaptation or against pests and pathogens is evident. At the same time, some fungi are necessary to tree health as symbiotic or endophytic partners. It is possible that differences in the microbiota will influence the processes leading to disease outbreak, as fungal pathogens need to compete with other endophytes for key metabolites in the same niche. The “insurance hypothesis” in the field of evolutionary ecology suggests that high diversity maintains the overall integrity of an ecosystem while biotic and abiotic environmental conditions change. Thus, the hidden biodiversity of microbiomes may enhance the tolerance of the trees to fungal pathogens and furthermore, beneficial microbiomes could act as biocontrol agents in integrated pest management of the future.

To date, plant-fungal interactions have rarely been studied on the mycobiome level. Especially comparative investigations of fungal communities in distinct habitats and under distinct climate regimes have been sparse. It is not well-known how the host genotype and physiological stage modifies the endophytes as a part of tree mycobiome. All invading fungal organisms need to deal with host defenses. Interestingly, trees have evolved diverse mechanisms to protect themselves—they seem to be able to separate harmful microbes from beneficial symbionts. However, the versatility of fungal life strategies has questioned our understanding of plant mycobiome function. May friends turn to foes?

Within this topic, four articles have been published that complemented our knowledge of the importance of the hidden biodiversity. Qu et al. analyzed the fungal community at a regional scale in boreal forests. The three unmanaged subarctic, northern and southern boreal forests, formed distinct fungal community structures. Compared to the two northern locations, the southern boreal forest harbored greater abundance of *Zygomycota, Lactarius, Mortierella, Umbelopsis*, and *Tylospora*, in which aspect there were no differences between the two northern forests. *Cortinarius, Piloderma*, and *Suillus* were the core fungal genera in the boreal Scots pine forest. Functionally, the southern boreal forest harbored a greater abundance of saprotroph, endophytes and fungal parasite-lichen, whereas ectomycorrhizal fungi were more common in the northern boreal forests. Moreover, the pathotroph and wood saprotrophs were commonly present in all regions. The three locations formed two distinct fungal community functional structures, by which the southern forest was clearly different from the two northern forests, suggesting a distance–decay relationship *via* geographic location. The study highlighted the core fungal community composition and potential functional groups in three forests dominated by Scots pine (*Pinus sylvestris* L.). It appears that some fungal taxa are generalists across geographic locations.

Beech trees (*Fagus sylvatica*) are valuable tree species of European forests. The topic presents two studies focusing on microbiome of beech. Siddique et al. evaluated the effect of plant organ and growth habitat mycobiomes of buds and twigs of two young healthy mountain beech stands. Richness and diversity indices correlated (*p* < 0.05), and mycobiomes did not differ between habitats in the current study. Species richness and diversity were higher in twigs compared to spring buds. Interaction network analyses suggested that competitive exclusion in mycobiomes may be the predominant ecological interaction within twigs. It appears that plant organs may set boundaries for endophytic communities directly affecting colonization success and indirectly by facilitating competitive interactions between the fungi. Overall, the plant organ type had higher effect on the microbiome than growth site.

Vitality loss is a beech disease of complex symptoms frequently observed in Europe. Langer and Buβkamp focused on the interaction of microbiome and tree health with culture-based isolation methods, in planta inoculations and fungal identification using ITS-barcode and morphological characters. Endophytes of healthy beech saplings were isolated. Beech saplings were inoculated with selected fungal pathogens and wood inhabiting fungi (*Hypocreales, Botryosphaeriales*, and *Xylariales*) originating mainly from symptomatic beech. *Botryosphaeria corticola* caused most severe canker symptoms in the experiment and it might be a severe pathogen combined with of abiotic stress in changing climate. *Neonectria coccinea* appears to haves a key role in killing the bark and causing the loss of vitality in beech.

Another recent health problem of European hardwoods, ash decline, has severely affected the ash populations in Europe. In its native range, invasive *Hymenoscyphus fraxineus* is either harmless endophyte or leaf saprophyte. Hietala et al. defined mycobiome and hyphal growth pattern of European ash leaves and report for the first time the natural colonization by *Hymenoscyphus albidus*. Their aim was to clarify the behavior of native *H. albidus* and its spatial and temporal niche overlap with the invasive *H. fraxineus* in living tissues of European ash. The necrosis area of the ash leaves had higher number of functional group endophyte/necrotrophy than compared to healthy tissue. The ITS rDNA from ash leaves revealed that fungal species composition varied between the study sites, but species *Cladosporium* sp., *Dioszegia* sp., and *Phoma* sp. was found in each site. Interestingly, *H. fraxineus* was found from two sites where native *H. albidus* was not present. Both species can turn to pathogenic in late autumn.

In conclusion, this topic accomplished the understanding that composition of microbiomes depends on plethora of biotic and abiotic factors such as such as tree health, plant organ or tissue, and growth habitat. Also accumulating evidence suggests that plants harbor diverse mycobiota, which has profound effects on their health.

## Author contributions

All authors listed have made a substantial, direct, and intellectual contribution to the work and approved it for publication.

